# Supplementary material for: Theoretical investigation of a genetic switch for metabolic adaptation
Source: PLoS One. 2020 May 7;15(5):e0226453. doi: 10.1371/journal.pone.0226453 (PMC7205307; doi:10.1371/journal.pone.0226453)
Supplement: S1 Text — Discussion of simplifications in the model, parameter estimation, elaborations on the results, and the chemical master equation of this circuit. Experimental materials and methods. (PDF) [file pone.0226453.s001.pdf]

Supporting information for "theoretical investigation of a genetic switch for metabolic adaptation"

Kathrin S. Laxhuber[1], Muir J. Morrison[2], Griffin Chure[3], Nathan M. Belliveau[4], Charlotte Strandkvist[5], Kyle L. Naughton[6], Rob Phillips[2,3*]

1 Department of Chemistry and Applied Biosciences, ETH Zurich, 8093 Zurich, Switzerland
2 Department of Physics, California Institute of Technology, Pasadena, CA, USA
3 Division of Biology and Biological Engineering, California Institute of Technology, Pasadena, CA, USA
4 Howard Hughes Medical Institute, University of Washington, Seattle, WA, USA
5 Department of Systems Biology, Harvard Medical School, Boston, MA, USA
6 Department of Physics and Astronomy, University of Southern California, Los Angeles, CA, USA

∗ phillips@pboc.caltech.edu

A Model choices, simplifications and assumptions

This part of supporting information text offers a more detailed discussion of the assumptions that were made in the model and explains the choices that were made.

Simplistic modeling of transcription. The precise mechanism at the promoter level is not the key feature of this system, and we expect the same qualitative results for slightly different transcription models. This work aims to gain an intuition from the results, and the more unknown parameters there are, the harder this becomes. Furthermore, the mechanistic details of transcription are not entirely known and would make the model too complex: for example, transcription probably is not a Poisson process, but comes in bursts. Additionally, very little is known about the function of the *xapAB* promoter. For these reasons, we do not model every detail, but focus on the most relevant features of the system, while still trying to be physically correct.

Firstly, the equilibrium assumption is not completely accurate, but has proven successful in previous work. An example for this is the very detailed analysis of a gene with one repressor in [1]. Secondly, different binding energies of XapR to its two binding sites would change the equations as follows: the factor $\left(\frac{1}{K_{\mathrm{XapR}}}\right)^2$ would become $\frac{1}{K_{\mathrm{XapR},1}K_{\mathrm{XapR},2}}$ and the factor $\frac{2}{K_{\mathrm{XapR}}}$ would be replaced by $\frac{K_{\mathrm{XapR},1}+K_{\mathrm{XapR},2}}{K_{\mathrm{XapR},1}K_{\mathrm{XapR},2}}$. For most choices of K_{XapR}, this does not give the exact same results quantitatively, but we do observe the same qualitative outcome (see also next paragraph). Thirdly, we consider all but one state as inactive, which is not strictly correct. Still, as mentioned in the main text, experiments suggest that this is reasonable. The fourth main simplification, which is the independence of polymerase and XapR binding, will be discussed in a separate subsection below.

In Fig 1, we have plotted phase portraits where some of the above assumptions have not been made. For that, we have chosen some extreme values for the new parameters to demonstrate that these simplifications do not have a significant influence. The two additional transcription rates are large compared to experimental observations. Moreover, the chosen values of $[\mathrm{XapR}]_{\mathrm{R},i}$ correspond to a difference of two orders of magnitude between $K_{\mathrm{XapR},1}$ and $K_{\mathrm{XapR},2}$. The difference between Oid and O3 in *lac* are two to three orders of magnitude [2]. The values were chosen such that $K_{\mathrm{XapR},1}K_{\mathrm{XapR},2} = K^2_{\mathrm{XapR}}$. Unsurprisingly, this is necessary to not observe large deviations from the original equations. The results lead to the conclusion that, for the purposes of this paper, the aforementioned simplifications are appropriate.

Activator and polymerase interaction. A mechanistically more precise model of activation would include an interaction energy between polymerase and XapR. In that case, p_{active} cannot be factored into separate terms for the polymerase and the XapR binding probability. However, the precise activation mechanism is unknown.

Moreover, introducing an interaction energy between polymerase and XapR would make the equations overly complex. This would not only make factorization impossible, it would also be just as consequential to have different dissociation constants and additional rates as in the previous paragraph. This leads to a huge number of terms and parameters because of different interaction energies for every state, which can be foreseen from the states and weights shown in the main text. The precise transcription mechanism is not one of the central parts of the circuit we are investigating (see also discussion in the previous paragraph). Thus, making it the by far most complex and precise part of the equations would be unreasonable and hinder the understanding of the more relevant components of the system.

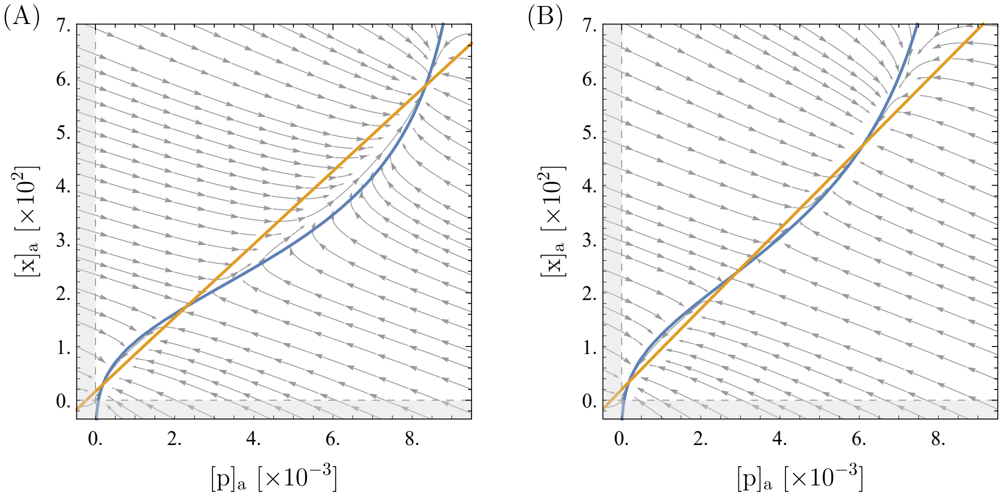

Fig 1. Results for different models of transcription. The parameters are the same as the ones that were used for all other phase portraits in this report and that are listed in a table in the main part. In (A), the phase portrait after addition of non-zero transcription rates to all other polymerase-bound states is shown (third assumption lifted). The new parameter values are $r_{\mathrm{I}} = 10^{-2} r_{\mathrm{p}}$ for the transcription rate when only one XapR is bound and $r_0 = 10^{-3} r_{\mathrm{p}}$ for the basal rate. In (B), the phase portrait after introducing different XapR dissociation constants can be seen (second assumption lifted). The new parameter values are $[\mathrm{XapR}]_{\mathrm{R},1} = 10$ and $[\mathrm{XapR}]_{\mathrm{R},2} = 0.1$.

Table 1. States and weights for a more precise model of transcription.

XapR	Pol.	Transcription	Weight
none	no	0	1
first	no	0	w_{X1}
second	no	0	w_{X2}
both	no	0	$w_{\mathrm{X1}} w_{\mathrm{X2}} e^{\Delta\epsilon_{\mathrm{coop}}}$
none	yes	r_0	w_{p}
first	yes	r_{X1}	$w_{\mathrm{p}} w_{\mathrm{X1}} e^{\Delta\epsilon_{\mathrm{X1}}}$
second	yes	r_{X2}	$w_{\mathrm{p}} w_{\mathrm{X2}} e^{\Delta\epsilon_{\mathrm{X2}}}$
both	yes	r_2	$w_{\mathrm{p}} w_{\mathrm{X1}} w_{\mathrm{X2}} e^{\Delta\epsilon_{\mathrm{X1}}+\Delta\epsilon_{\mathrm{X2}}+\Delta\epsilon_{\mathrm{coop}}}$

The first column shows which XapR sites are occupied in this state, the second one whether polymerase is bound to the promoter, and the third the transcription rate. In the weights, the notation is as follows: w_{X1} is the weight for XapR binding to the first binding site on the promoter, w_{X2} is that to the second site, w_{p} is the weight of polymerase binding to the promoter, $e^{\Delta\epsilon_{\mathrm{X1}}}$ is the interaction energy between one XapR on the first binding site and the polymerase that is bound, $e^{\Delta\epsilon_{\mathrm{X2}}}$ is the same for the second binding site, and finally, $e^{\Delta\epsilon_{\mathrm{coop}}}$ is the interaction energy of the two XapR at both binding sites as in the main part.

Furthermore, we do not expect our approximation to have a large influence: the interaction energy will make the doubly bound state dominate, and the polymerase-XapR interaction should be weak in the singly bound state, because very weak activation is observed in the experiment when one of the XapR binding sites is removed (see section E in this supporting information text). If $e^{\Delta\epsilon_{X1}}$ and $e^{\Delta\epsilon_{X2}}$ are small, we have $w_p w_{X1} e^{\Delta\epsilon_{X1}} \approx w_p w_{X2} e^{\Delta\epsilon_{X2}} \approx w_p w_X$. If, in addition, the transcription rates for the singly bound states are much smaller than those for the doubly bound state, we can neglect the singly bound states in the nominator of p_{active} (which, as argued in the previous paragraph "Simplistic modeling of transcription", is a good assumption). Thereby, we can obtain the same form of p_{active} that we are working with in the main part by including the interaction between polymerase and XapR into ϵ_{coop}.

Working with only one protein species. The proteins are transcribed together, and thus, their rate of transcription and mRNA decay should be the same. The protein decay is assumed to be dominated by cell division, making the decay rates roughly equal as well. What could differ is the translation rate. Changes in this rate rescale the protein synthesis rate by the same factor and thus, in steady-state, change the total number of proteins. The only other place where the proteins appear in the ODE's is in their respective Michaelis-Menten term, and the latter is proportional to $k_{\text{cat}}[\text{E}]_0$. Hence, different numbers of XapA and XapB can be accounted for by changing their respective values of k_{cat}.

Exponential protein decay. The protein decay should be dominated by dilution through cell division, which is a discrete process. However, the cell grows before division, resulting in a continuous dilution in concentration. Furthermore, replication leads to a varying number of gene copies and thereby also a varying transcription rate. Trying to capture all these mechanistic details would lead to an overcomplicated model. Treating the protein decay as a Poisson process has produced correct results in past research, which is why we expect this assumption to be reasonable.

Kinetics of XapB, NupC, and NupG. All statements in this paragraph are based on data from [3]. Xanthosine entering the cell consists of two steps. In a first transport step, xanthosine passes the outer membrane through porins like Tsx, OmpF and OmpC. This does not seem to be a rate-limiting step. Then, XapB actively transports it across the inner membrane, powered by the proton motive force. There are two other active nucleoside transporters that seem to be transporting xanthosine with a very low affinity: NupC and NupG. It was found that Δ*nupC* Δ*nupG* strains cannot grow on xanthosine. Hence, these seem to be necessary to "start" the system by importing small amounts of xanthosine that then activate *xapB*, and there appears to be no other significant way in which xanthosine can enter the cell.

As mentioned in the main text, we assume the kinetic scheme of all three transporters to be similar to that of *lac* permease (as it is described in [4]), which is discussed in more detail in the next subsection. One transporter of this type can do two things to change the intracellular xanthosine concentration [x]: it can actively transport one xanthosine into the cell, powered by the proton gradient, or it can transport one out of the cell, against the proton gradient. For net transport, a proton and a substrate need to bind on one side of the membrane and detach from the transporter on the other side of the membrane before the transporter goes back to the first side again (see next subsection). Because of the proton gradient, influx is overall thermodynamically more likely than export at low intracellular xanthosine concentrations, which leads to a

net influx. For much higher intra- than extracellular xanthosine concentrations, the difference in the chemical potential of xanthosine across the membrane can dominate that of the protons and there is net efflux.

We model influx and efflux separately and use Michaelis-Menten kinetics for XapB (this is discussed in more detail in the next subsection). For the turnover rate and Michaelis constant for influx we write $k_{\mathrm{b,i}}$ and $K_{\mathrm{b,i}}$, respectively, and $k_{\mathrm{b,e}}, K_{\mathrm{b,e}}$ for efflux. These parameters implicitly include the proton gradient.

For NupC and NupG, we approximate the transport reactions as linearly dependent on the substrate. This should be appropriate, since their affinity for xanthosine is so low that it cannot even be measured properly [3], which makes K_{M} much larger than c or [x] will ever be. Thus, the general $k_{\mathrm{cat}}[\mathrm{E}]_0 \frac{[\mathrm{S}]}{K_{\mathrm{M}}+[\mathrm{S}]}$ can be approximated as $\frac{k_{\mathrm{cat}}}{K_{\mathrm{M}}}[\mathrm{E}]_0[\mathrm{S}]$. In our equations, $\frac{k_{\mathrm{cat}}}{K_{\mathrm{M}}}[\mathrm{E}]_0$ is denoted by k_{nup} for influx, and the efflux rate is written as ξk_{nup}. For the XapB kinetics, no such approximation can be made, because the xanthosine concentration in the dynamic system can range from far below the respective K_{M} value to far above.

The kinetics of *lac* permease. All transporters in our system are driven by the proton gradient [3], and we assume their kinetic scheme to be like that of *lac* permease. This scheme is shown schematically in Fig 2. Even though it includes several steps, the essential part is substrate binding, conformational change of the transporter, and substrate release on the other side of the membrane. We assume the proton gradient across the membrane to be constant and proton binding and unbinding to the transporter to be fast. Hence, the latter does not need to be included explicitly in the kinetic model (it is implicitly part of the turnover rate).

Substrate release should be much faster than conformational change, which is analogous to ignoring the "EP" state in Michaelis-Menten (enzyme with product bound, here the conformationally changed transporter with substrate still bound). Also, it should be reasonable to neglect the immediate backwards reaction (before proton release), and thus, Michaelis-Menten kinetics is obtained. Data from [5] and [6] actually show that, at a physiologically reasonable proton gradient, both, influx and efflux, can be described by the Michaelis-Menten equation very well.

What is neglected in this modeling is the fact that the transporter needs to release the proton and undergo a conformational change again before being able to conduct the same reaction again. However, by using experimentally determined apparent values to estimate the kinetic constants, this is already included in these parameter values. Another simplification we make is to treat influx and efflux as two independent reactions instead of thinking of it as one reversible kinetic process. This seems reasonable, though, because the transporter needs to lose its proton between reactions in order to obtain a net transport. If the transporter has for example done steps 1-6 in Fig 2, it can from there on really enter two entirely different reactions: it can do its conformational change in its empty state (steps 7 and 8) to then transport a substrate again (steps 1-6) or it can bind a new proton and a new substrate directly and transport them back (steps 6-1). The former would give influx and the latter efflux, so the two of them can be modeled as separate reactions.

Passive transport across the membrane. Most small molecules diffuse passively through the membrane, but at physiological pH, xanthosine is mostly negatively charged and charged substances do not significantly diffuse across the membrane on their own. Nevertheless, from the $\mathrm{pK_a}$ of xanthosine we find that at physiological pH, about 1.5%

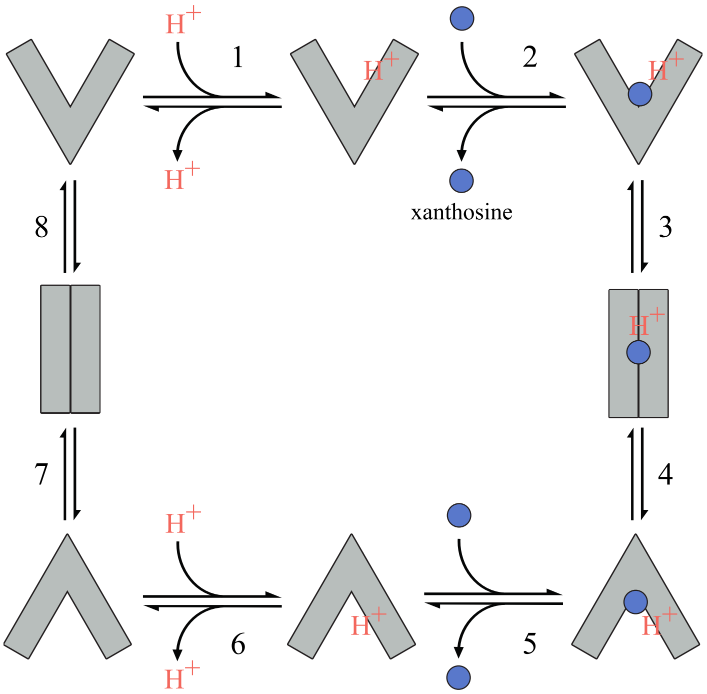

Fig 2. Kinetic scheme for transport by proton-driven pumps. The mechanism of lac permease as for example described in [4] is used as a model for xanthosine transport. The enzyme binds a proton and a substrate (steps 1 and 2), undergoes conformational change (steps 3 and 4), and then releases substrate and proton on the other side of the membrane (steps 5 and 6). When the enzyme is empty, it can change its conformation again to repeat the transport (steps 7 and 8). Instead, it could also bind a new proton and substrate and transport them in the opposite direction (steps 6, 5, 4, 3, 2, 1). Alternatively, it could also keep the proton and just cycle back and forth, exchanging substrates from outside and inside the cell (steps 5, 4, 3, 2).

of it is uncharged and could pass the membrane. This could be modeled as follows:

$$\left(\frac{\mathrm{d}[\mathrm{x}]}{\mathrm{d}t}\right)_{\mathrm{diff}} = JA = p(c - [\mathrm{x}])A = \underbrace{pA}_{\text{rate}}(c - [\mathrm{x}]) \tag{1}$$

Here, J is the flux through the membrane, which follows directly from Fick's first law (if steady-state diffusion is assumed). The other new parameters are the membrane area A and the membrane permeability coefficient p of the diffusing substrate.

The diffusion rate $k_{\mathrm{d}} = pA$ can be estimated with the help of Overton's rule. In our model, we find that this passive diffusion alone (i.e. without NupC and NupG) is enough to obtain bistability in the deterministic analysis as well as bimodality in the stochastic simulations. However, experiments show that $\Delta nupC$ $\Delta nupG$ strains cannot grow on xanthosine [3]. This suggests that passive diffusion alone is not enough to make the cells switch. Experiments with cells in minimal media containing some glucose and a xanthosine concentration in the bimodal regime show that the switching time can be several generations. The same is observed in our simulations. The xanthosine concentration that was used for the double knockout experiments, where no glucose was present, is just above the switching threshold, which is when switching is slow. For this reason, we suspect that without NupC and NupG and with only xanthosine as an energy resource, the cells starve before they have switched. This probably is the reason why passive diffusion is, in reality, not enough to observe switching. NupC and NupG can probably keep the cells going at very low intracellular xanthosine concentrations. Because of the active transport, they can accumulate a higher intracellular than extracellular concentration of xanthosine.

Since NupC and NupG transport seems to be much more significant than diffusion, we do not include the latter explicitly in our model. However, because it takes the form given in Eq 1, it can easily be accounted for implicitly by slightly changing the values of k_{nup} and ξ.

Other mechanisms for xanthosine degradation. There probably are some ways in which xanthosine is degraded, other than by XapA. Otherwise, initially uninduced cells that are transferred to only xanthosine as an energy source might not survive until they have switched. As mentioned previously, it was found that they do [3], so they need to be able to metabolize xanthosine somehow. Nevertheless, these degradation pathways cannot have a big effect: as argued later on when estimating the Nup parameters, the intracellular xanthosine concentration in the uninduced state must be a bit larger, but not too much larger, than the extracellular one. Thus, degradation of xanthosine has to be small because import through Nup is weak. Due to this, it should be appropriate to linearize the equations describing these effects. Then, they can simply be accounted for in the term for Nup transport by making slight changes in the values of ξ and k_{nup}. It could, however, also be that the basal level of XapA is enough to obtain the small degradation effect without any other pathways.

B Parameter estimation

In this section we present a detailed estimation of the values of all nondimensional parameters. Two dimensionful parameters appear in most nondimensional ones, namely γ_p and K_a. For this reason, we start by estimating these.

The protein decay rate γ_p: Protein decay is generally dominated by dilution through cell growth and division. The average doubling time of *E. coli* is 20-30 minutes. We take this as an estimate for the protein half life, which leads us to a decay rate of $\gamma_\mathrm{p} = \frac{\ln 2}{t_{1/2}} \approx 5^{+2}_{-3} \cdot 10^{-4}\ \mathrm{s}^{-1}$. Note that these upper and lower bounds correspond to half-lifes between 15 and 60 minutes, which should be reasonable. For the following nondimensionalization, we work with a fixed value of $\gamma_\mathrm{p} = 5 \cdot 10^{-4}\ \mathrm{s}^{-1}$.

The Michaelis-Menten parameters for XapA (K_a, k_α): The Michaelis-Menten parameters of XapA kinetics have been measured in two independent publications [7,8]. Both have found similar values and we conclude $K_\mathrm{a} \approx 6 \pm 3 \cdot 10^{-5}$ M and $k_\mathrm{a} \approx 10^{-1\pm0.8}\ \mathrm{s}^{-1}$. This gives $k_\alpha \approx 10^{2\pm0.8}$. We choose to work with $K_\mathrm{a} = 5 \cdot 10^{-5}$ M in the following.

The extracellular xanthosine concentration $[\mathrm{c}]_\mathrm{a}$: In the experiment, switching occurred if cells were grown in a solution with a concentration of xanthosine of roughly a few mM [3]. According to these experimental observations, the interesting range of xanthosine should be $[\mathrm{c}]_\mathrm{a} \in [0, 10^3]$ (nondimensionalized), which corresponds to $\mathrm{c} \in [0, 5 \cdot 10^{-2}]$ M. However, we are careful because there are issues with the solubility of xanthosine.

The MWC parameters $K_{\chi\mathrm{A}}$, K_IA, and $\Delta\epsilon_\mathrm{x}$: The three MWC parameters are the energy barrier between the active and inactive state of XapR and the equilibrium constants of xanthosine binding to XapR in the two states. In Fig 3, the induction curve as a function of the nondimensional parameters is shown. We assume that evolution has tuned the induction curve such that less than 10% of XapR is active in the absence of xanthosine, and more than 90% is active for [x] approaching infinity. This is a relatively weak assumption, otherwise XapR would not really be fulfilling its job. It helps us by putting a constraint on K_IA and $\Delta\epsilon_\mathrm{x}$:

$$p_\mathrm{active}([\mathrm{x}]_\mathrm{a} = 0) = \frac{1}{1 + e^{\Delta\epsilon_\mathrm{x}}} < 0.1$$
$$1 - p_\mathrm{active}([\mathrm{x}]_\mathrm{a} \to \infty) = \frac{e^{\Delta\epsilon_\mathrm{x}}}{K_\mathrm{IA}^2 + e^{\Delta\epsilon_\mathrm{x}}} < 0.1$$

Thus,

$$\Delta\epsilon_\mathrm{x} > 2.2 \qquad \text{and} \qquad K_\mathrm{IA} > 3e^{\Delta\epsilon_\mathrm{x}/2}.$$

A further constraint is found from $K_\mathrm{IA} = \frac{K_\mathrm{xI}}{K_\mathrm{xA}} = e^{\beta\Delta E}$, where ΔE is the energy difference between xanthosine binding to the two states. It is unlikely that this energy difference is more than 7 $k_\mathrm{B}T$, which corresponds to roughly three strong hydrogen bonds. This gives us an upper bound $K_\mathrm{IA} < 10^3$ and thereby $\Delta\epsilon_\mathrm{x} < 10^1$. For $K_\mathrm{IA} = 10^2$, the upper bound is $\Delta\epsilon_\mathrm{x} < 7$. This gives us a range of $K_\mathrm{IA} \approx 10^1 - 10^3$ and $\Delta\epsilon_\mathrm{x} \approx 2 - 10$ or, more precisely, $\Delta\epsilon_\mathrm{x} \approx 2$ to $\ln\left(\frac{1}{9}\left(K_\mathrm{IA}\right)^2\right) \approx 2\left(\ln\left(K_\mathrm{IA}\right) - 1\right) < 12$. Compared with other systems, these values do seem reasonable.

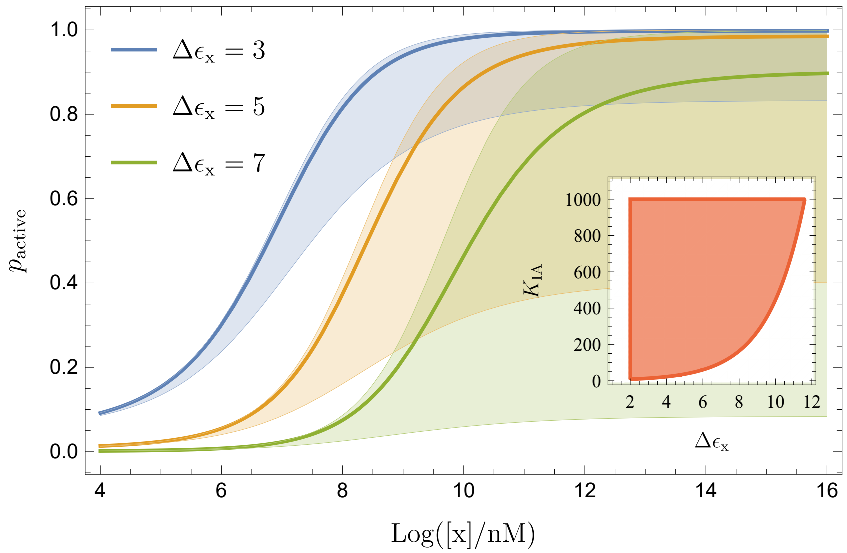

Fig 3. Induction curves given by MWC model. The main plot shows the probability of XapR being in the active state as a function of the logarithm of the xanthosine concentration for $\Delta\epsilon_x$ as shown, $K_{\chi A} = 10^2$, and $K_{IA} = 10^2$. The shaded areas indicate where the curve lies for changes in K_{IA} of one order of magnitude. Changing $K_{\chi A}$ by one order of magnitude, on the other hand, shifts all the curves by one order of magnitude in the corresponding direction to the left or right. The inset shows how the allowed regimes of $\Delta\epsilon_x$ and K_{IA} depend on each other. This explains why for $\Delta\epsilon_x = 7$ and $K_{IA} = 10$ (lower green line in the main plot), p_{active} at infinite xanthosine concentrations becomes far too low.

To find $K_{\chi\mathrm{A}}$ we furthermore assume that the steep part of the induction curve lies in the biologically relevant xanthosine regime, which estimated experimentally, see the paragraph about $[\mathrm{c}]_\mathrm{a}$ above. Again, this is a rather weak assumption. As can be seen from Fig 3, this assumption sets the value range of $K_{\chi\mathrm{A}}$. The line for $\Delta\epsilon_\mathrm{x} = 5$ in Fig 3 is at the experimentally expected position in the xanthosine regime. It becomes clear from the figure that the position of the steep range is strongly affected by $\Delta\epsilon_\mathrm{x}$, and a change of 1 in the latter leads to roughly a change of 10^1 in the former. Thus, we estimate $K_{\chi\mathrm{A}} \approx 10^{2\pm1} \cdot 10^{\Delta\epsilon_\mathrm{x}-5}$ and thereby finish our estimation of the MWC parameters.

The XapR concentration $[\mathrm{XapR}]_\mathrm{R}$**:** It is experimentally known that the XapR copy number is very low, on the order of 10^{-8} M (10^1 molecules per cell) [9]. We estimate the XapR – DNA dissociation constant from the values for *lac* that were found in [2] and obtain $10^{-8\pm2}$ M, where we have added one additional order of magnitude as insecurity to the upper and lower bound. This gives $[\mathrm{XapR}]_\mathrm{R} = \frac{[\mathrm{XapR}]_\mathrm{tot}}{K_\mathrm{XapR}} \approx 10^{0\pm2}$.

The XapR cooperative energy $\Delta\epsilon_\mathrm{coop}$**:** Any cooperative energy should come mainly from an interaction between the two XapR molecules. It would be surprising if it was larger than 10 $k_\mathrm{B}T$, which corresponds to roughly 4 strong hydrogen bonds. Hence, we estimate $\Delta\epsilon_\mathrm{coop} \approx 0 - 10$.

The transcription rate parameter ρ_m**:** The transcription rate $r_\mathrm{m}\frac{[\mathrm{P}]}{[\mathrm{P}]+K_\mathrm{P}}$ in the presence of two activator molecules should be on the order of $10^{-2\pm1}$ nM s^{-1}, thus $\rho_\mathrm{m} := \frac{r_\mathrm{m}}{\gamma_\mathrm{p} K_\mathrm{a}} \frac{[\mathrm{P}]}{K_\mathrm{P}+[\mathrm{P}]} \approx 10^{-3\pm2}$. Note that the units are nM s^{-1} and not just s^{-1} because the corresponding rate equation is of the form $\frac{\mathrm{d[m]}}{\mathrm{d}t} = r \cdot p$ where the probability p is dimensionless and the left-hand side has dimensions of mRNA concentration per time.

This estimate comes from the assumption that this rate should be roughly $10^1 - 10^2$ times larger than a normal basal rate (of other genes), for which common values are around $10^{-3\pm1}$ nM s^{-1} [10]. Because experiments show that compared to the activated promoter, the basal transcription rate is almost zero for *xapAB* (probably due to the weak polymerase binding site), this actually means a 10^2- to 10^3-fold increase in the transcription rate due to activation by XapR.

Keep in mind that the rate includes not only the effect of XapR on the pure speed of transcription, bt also any effect of XapR on the polymerase dissociation constant. Still, we do not expect higher values such as 10^0 nM s^{-1} since this is roughly the rate for transcription of rRNA and the latter should be significantly faster. To further validate our estimate, we can compare it to measured values for *lac*, which should be roughly the same because the gene function is similar. The transcription rate for *lac* in the absence of any repression that was found in [11] corresponds well with our estimate.

The mRNA decay rate parameter γ_mp**.** The mRNA life time is usually on the order of 1 to 8 minutes which gives a decay rate of $\gamma_\mathrm{m} \approx 10^{-3} - 10^{-2}$ s^{-1}. Thus, we have $\gamma_\mathrm{mp} := \frac{\gamma_\mathrm{m}}{\gamma_\mathrm{p}} \approx 10^{1\pm0.5}$.

The translation rate parameter ρ_p**.** The translation rate should be on the order of $r_\mathrm{p} \approx 10^{-2} - 10^{-1}$ s^{-1} [10]. This gives $\rho_\mathrm{p} := \frac{r_\mathrm{p}}{\gamma_\mathrm{p}} \approx 10^{2\pm0.5}$.

The Michaelis-Menten parameters for XapB ($k_{\beta,\mathrm{i}}$**,** $K_{\beta,\mathrm{i}}$**,** $k_{\beta,\mathrm{e}}$**,** $K_{\beta,\mathrm{e}}$**):** To estimate the parameters for both influx and efflux, we need data from experiments where these two processes have been measured independently. Lactose permease, which we

expect to function in a similar way as XapB, is comparatively well studied such that these rates have been measured in multiple ways and for different conditions. Some data from several publications is collected in [6] and we will base our estimates on this and the corresponding references.

Comparing the effective rates and Michaelis constants of lactose permease and the nucleoside transporters NupC and NupG shows that their orders of magnitude are similar: from [12, 13] we find $k_{\text{cat}} \approx 10^1\ \text{s}^{-1}$, $K_{\text{M}} \approx 10^1 - 10^2\ \mu\text{M}$ for LacY and from [3, 14, 15] $k_{\text{cat}} \approx 10^2\ \text{s}^{-1}$, $K_{\text{M}} \approx 10^0 - 10^1\ \mu\text{M}$ for pyrimidines through NupC and NupG. Note that especially for Nup, these are only rough measurements, and it is known that NupC behaves different with purines (thus also with xanthosine). Given these values, we expect lactose permease to be similar enough to nucleoside transporters so that we can use it as a starting point for our estimation of the XapB kinetic values.

We use the measured influx values that are summarized in [6] for a membrane potential of 100 mV and obtain $k_{\text{b,i}} \approx 10^{1\pm1}\ \text{s}^{-1}$ and $K_{\text{b,i}} \approx 10^{-4\pm2}$ M. Here, the uncertainty is estimated from the distribution of values of many kinds of enzymes that is presented in [10]. The bounds also reflect the difference between LacY and Nup that was found above. For the dimensionless quantities, this gives $k_{\beta,\text{i}} \approx 10^{4\pm1}$ and $K_{\beta,\text{i}} \approx 10^{1\pm2}$.

For the efflux parameters, we proceed similarly and obtain $k_{\text{b,e}} \approx 10^{0\pm2}\ \text{s}^{-1}$ and $K_{\text{b,e}} \approx 10^{-3\pm3}$ M, therefore $k_{\beta,\text{e}} \approx 10^{3\pm2}$ and $K_{\beta,\text{e}} \approx 10^{2\pm3}$. Because of disagreement in literature, difficulty in measuring these parameters, and a lack of knowledge about what value range should be expected, we put larger error bounds than for the influx.

The non-specific transport parameters k_η, ξ: Estimating the rates of the Nup transporters for xanthosine is difficult since there are no experimental values that could be used as a reference. Because the rate cannot even be measured [3], it must be much lower than that of XapB. The kinetic parameters should, however, be such that the steady-state intracellular xanthosine concentration is significantly larger than the extracellular one. Otherwise, there would be no relevant difference to passive diffusion, which was shown to be insufficient for switching [3]. This essentially means $\xi < 1$, which on the other hand implies that the Nup rate is larger than or at least equal to the diffusion rate: If Nup transport were slower than diffusion, the latter would rule and ξ would be close to 1 (because this is the ξ value of diffusion).

In addition, we know from *lac* permease that influx is not much faster than efflux, which implies that ξ also cannot be too much smaller than 1. We guess $\xi \approx 0.8 \pm 0.1$. The number of Nup transporters per cell is roughly $10^2 - 10^3$ [9]. We expect $\frac{k_{\text{cat}}}{K_{\text{M}}}$ to be at least two or three orders of magnitude lower for Nup than for saturated XapB, where we have $\frac{k_{\text{b}}}{K_{\text{b}}} \approx 10^{5\pm3}\ \text{M}^{-1}\ \text{s}^{-1}$. These arguments set an estimated upper bound on the non-specific transport rate: $k_{\text{nup}} = \frac{k_{\text{cat}}}{K_{\text{M}}}[\text{E}]_0 \lesssim 10^{-3\pm3}\ \text{s}^{-1}$.

To find a lower bound, we estimate the diffusion rate. As described in Eq 1 in the previous section, the diffusion rate can be written as $10^{-2} \cdot p \cdot A$, where p is the permeability coefficient of the membrane for xanthosine, A is the total cell surface area, and 10^{-2} is the fraction of uncharged xanthosine at physiological pH. From the permeabilities of various substances that are collected in [10] and from the measured permeability values for purine nucleosides in [16], we estimate a permeability coefficient of $\tilde{p} = 10^{-8\pm3}\ \text{m s}^{-1}$. We write the tilde because this value is given in a convention where $\tilde{p} \cdot A = \frac{\text{d}x}{\text{d}t}$ with x being the number of xanthosine molecules and not the concentration. Since we work in concentrations, we divide by an estimated cell volume of $10^{-18}\ \text{m}^3$ and, with a cell surface area of $10^{-11}\ \text{m}^2$, find a diffusion rate of roughly $10^{-3\pm3}\ \text{s}^{-1}$. This sets the lower bound of k_{nup}.

Interestingly, the upper and lower bound turn out to be the same. Thus, we obtain the range $k_{\mathrm{nup}} \approx 10^{-3\pm3}$ s^{-1}, meaning $k_\eta \approx 10^{-1\pm3}$.

C Additional plots and explanations of the results

The 2D set of equations. If the mRNA concentration is set to steady-state as discussed in the main text, the dynamical system is reduced to the two equations shown below:

$$\frac{\mathrm{d[p]_a}}{\mathrm{d}\tau} = \rho \frac{\mathrm{[XapR]_{R,A}}^2 e^{-\Delta\epsilon_{\mathrm{coop}}}}{1 + 2\mathrm{[XapR]_{R,A}} + \mathrm{[XapR]_{R,A}}^2 e^{-\Delta\epsilon_{\mathrm{coop}}}} - \mathrm{[p]_a} \tag{2}$$

$$\frac{\mathrm{d[x]_a}}{\mathrm{d}\tau} = \left(k_{\beta,\mathrm{i}} \frac{\mathrm{[c]_a}}{K_{\beta,\mathrm{i}} + \mathrm{[c]_a}} - k_{\beta,\mathrm{e}} \frac{\mathrm{[x]_a}}{K_{\beta,\mathrm{e}} + \mathrm{[x]_a}} - k_\alpha \frac{\mathrm{[x]_a}}{1 + \mathrm{[x]_a}} \right) \mathrm{[p]_a} + k_\eta \left(\mathrm{[c]_a} - \xi \mathrm{[x]_a} \right) \tag{3}$$

$$\text{with} \quad \mathrm{[XapR]_{R,A}} = \mathrm{[XapR]_R} \frac{\left(1 + \frac{\mathrm{[x]_a}}{K_{\chi\mathrm{A}}}\right)^2}{\left(1 + \frac{\mathrm{[x]_a}}{K_{\chi\mathrm{A}}}\right)^2 + e^{\Delta\epsilon_{\mathrm{x}}} \left(1 + \frac{\mathrm{[x]_a}}{K_{\chi\mathrm{A}}} \frac{1}{K_{\mathrm{IA}}}\right)^2}.$$

Here, several of the parameters from the 3D system could now be combined into one by defining $\rho := \frac{\rho_{\mathrm{m}}\rho_{\mathrm{p}}}{\gamma_{\mathrm{mp}}}$.

Magnitude of flow in the phase portraits. The plots in the main part do not show the magnitude of the vector fields. For additional information, we show a 2D vector plot with the magnitudes in Fig 4.

Increasing $\mathrm{[c]_a}$. Fig 5 shows a family of nullclines for increasing extracellular xanthosine concentration. It shows the transition from monostability, to bistability, and back to monostability again.

Including transcriptional bursting. To test the robustness of our results to larger stochasticity (which is expected in the real biological system), we explored how the addition of burstiness in transcription altered our stochastic simulations. We assumed a geometric distribution for the burst size s, i.e. $p(s|\theta) = \theta \cdot (1-\theta)^{s-1}$ with mean $\theta^{-1} = 4$, which is comparable to or slightly larger than reported values [17]. We then truncate this distribution at a burst size of 10, meaning we did not consider larger bursts, leading to an actual mean of around 3.2. The transcription rate was rescaled such that the mean number of mRNA's produced remained the same.

We observe the same qualitative behavior but with stronger fluctuations around the mean and larger variation in the time to reach the steady-state mean. As an example, Fig 6A shows the time evolution of one run of the simulation with bursting for the same conditions (i.e. parameters and starting values) as the time evolution that was shown in the main text. In addition, the distribution of adaptation times can be seen in Fig 6B. Fig 7 shows the distribution obtained for the same and an additional condition (i.e. parameters and starting values) as for the distributions shown in the main text. The variance in the timescales and distributions is larger than without bursting. Furthermore, switching already happens for lower $\mathrm{[c]_a}$: in 7C, most cells are in the high expression state, whereas in the corresponding distribution in the main text, there were many uninduced cells, i.e. the first peak was higher. To obtain a bimodal distribution like in the main text, $\mathrm{[c]_a}$ now needs to be set to 17.5 instead of 18.5 as shown in 7B. This was to be expected, since the larger fluctuations make it easier for the system to jump across the middle fixed point.

Still, there are no big qualitative differences to the results without bursting. Thus, the assumptions we made that lead to less stochasticity than in the real biological

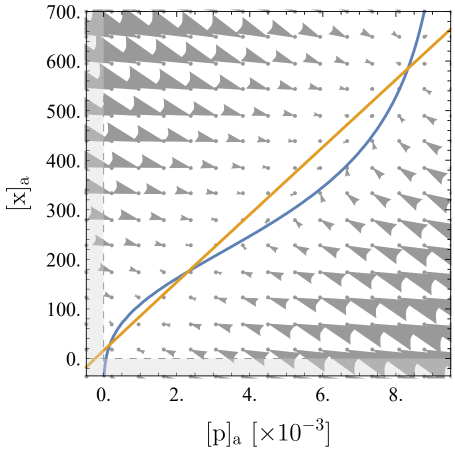

Fig 4. Vector plot of a standard case of bistability. The 2D phase portraits as a vector plot for for the standard set of parameters that was used in the main text.

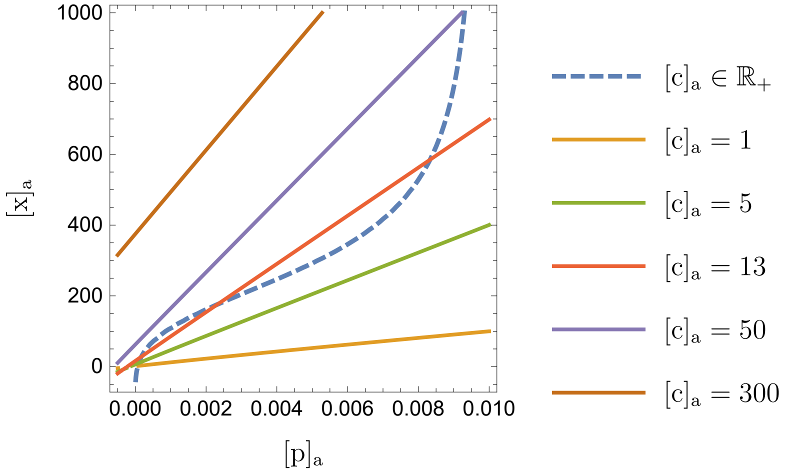

Fig 5. A family of curves for increasing $[c]_a$. The blue, dashed curve is the protein nullcline, which does not change with $[c]_a$. The other curves are the xanthosine nullclines for different values of the extracellular xanthosine concentration.

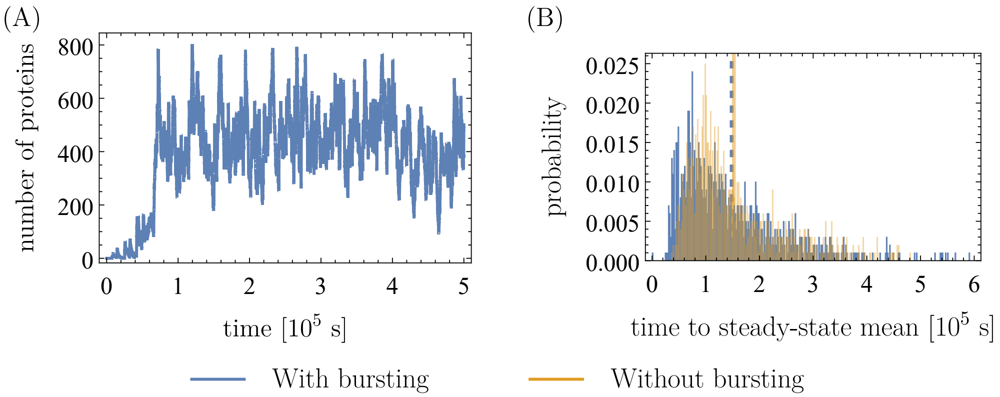

Fig 6. Time evolution and adaptation times from the stochastic simulation when transcriptional bursting is included. (A) shows the time evolution from one run of the simulation. The simulation was run once for $5 \cdot 10^5$ s and started at an mRNA, protein and intracellular xanthosine count of 0. The parameters that were used are the same as in the table in the main text, the only exception being the extracellular xanthosine concentration, which was chosen to be $[\mathrm{c}]_\mathrm{a} = 25$ (recall $[\mathrm{c}]_\mathrm{a} := \frac{c}{K_\mathrm{a}}$, $K_\mathrm{a} = 5 \cdot 10^{-5}\,\mathrm{M}$). (B) Blue shows the time until 90% of the protein concentration at the upper fixed point was reached in 1000 runs of the simulation (same conditions as (A)). The distribution from the main text (without bursting) is shown in orange for comparison. For both, the mean of the distribution is indicated by the line in the corresponding color.

system are not expected to have a very significant influence on our results, and the switch-like behavior seems to be stable to stochastic perturbations.

Hysteresis effects. In Fig 8, the results mentioned in the hysteresis part of the main text are shown. As mentioned there, the simulation was started with initially fully induced cells. At concentrations where no cells switched from the low to the high expression state before, cells here do not switch back to low expression. The extracellular xanthosine concentration (for the other parameters being as in the Table in the main text) needed to be as low as $[\mathrm{c}]_\mathrm{a} = 10$ to observe bimodality and $[\mathrm{c}]_\mathrm{a} = 9$ to have all cells in the low expression state (recall $[\mathrm{c}]_\mathrm{a} := \frac{c}{K_\mathrm{a}}$, $K_\mathrm{a} = 5 \cdot 10^{-5}\,\mathrm{M}$).

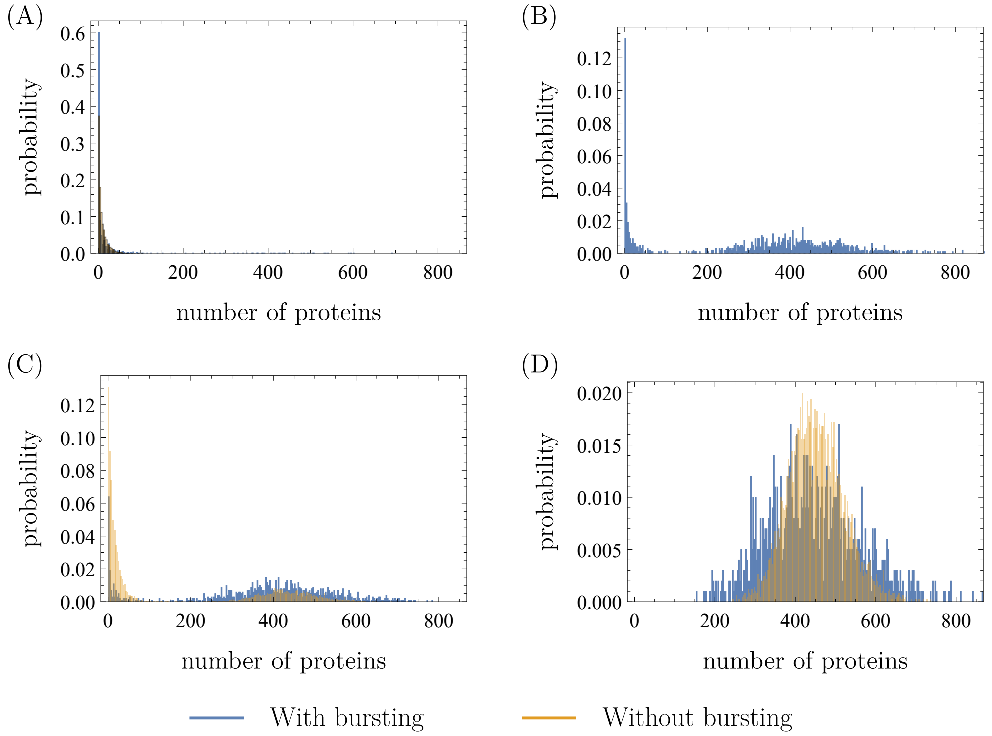

Fig 7. Distributions from the stochastic simulation when transcriptional bursting is included. Blue shows the distributions from the simulation with bursts, orange shows those without bursts from the main text for comparison. The following values of $[\mathrm{c}]_\mathrm{a}$ were used: (A) $[\mathrm{c}]_\mathrm{a} = 12$, (B) $[\mathrm{c}]_\mathrm{a} = 17.5$ (not shown in main text), (C) $[\mathrm{c}]_\mathrm{a} = 18.5$, and (D) $[\mathrm{c}]_\mathrm{a} = 25$ (recall $[\mathrm{c}]_\mathrm{a} := \frac{c}{K_\mathrm{a}}$, $K_\mathrm{a} = 5 \cdot 10^{-5}\,\mathrm{M}$). All other parameters are as in the table in the main text, and the simulation was run 1000 times for $10^6\,\mathrm{s}$ each (simulated time) and started at a mRNA, protein and intracellular xanthosine count of 0.

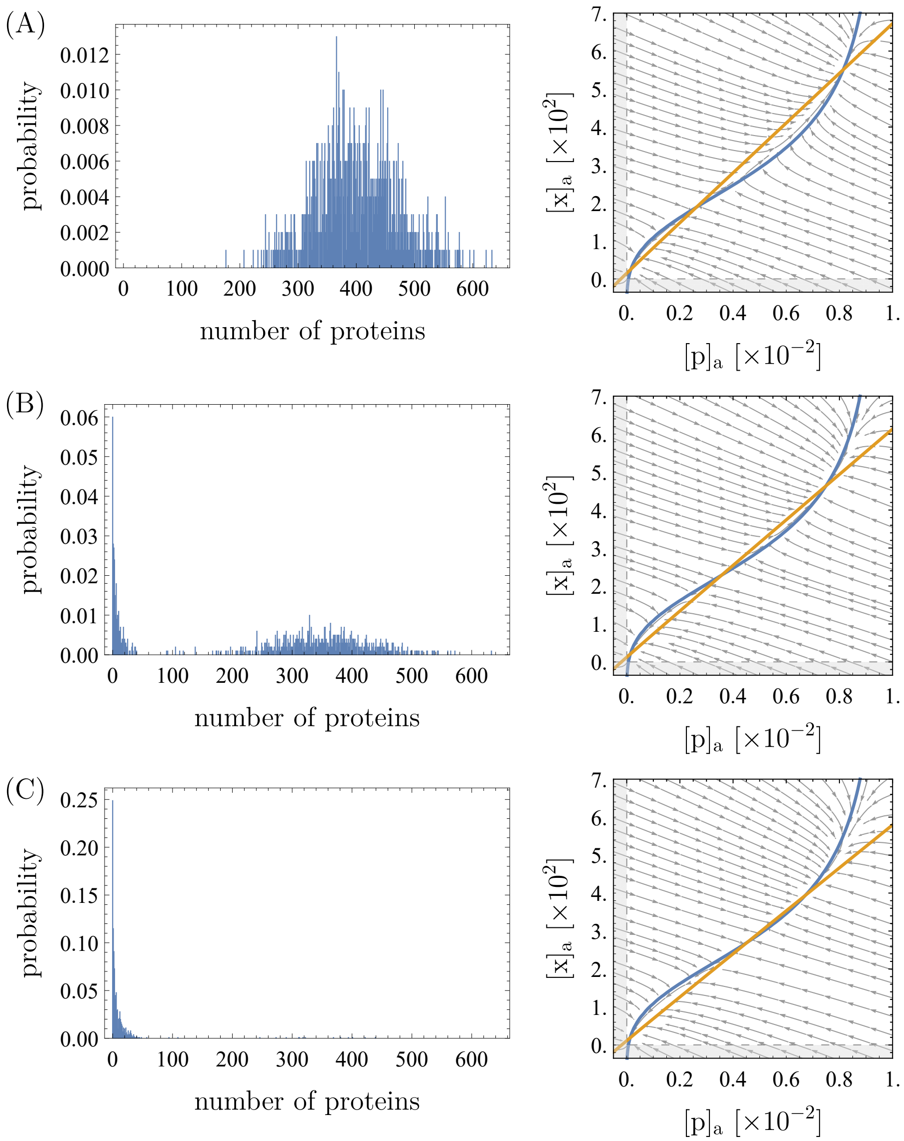

Fig 8. Decreasing $[\mathrm{c}]_\mathrm{a}$ in the fully induced system. Apart from $[\mathrm{c}]_\mathrm{a}$, the parameters are the same as in the table in the main text. For the distributions, the simulations were run 1000 times for 10^6 s each and started at the mRNA, protein and intracellular xanthosine counts of the high fixed point in the corresponding phase portraits. The values of $[\mathrm{c}]_\mathrm{a}$ are 12 in (A), 10 in (B), and 9 in (C). When comparing to the distributions in the main text where the simulation had initial concentrations of 0, a clear hysteresis effect can be observed.

D Chemical master equation

Here we provide some further details on our stochastic simulations by writing the chemical master equation for our model. The state of our system is described by the number of mRNA m, protein p, and xanthosine molecules x, and we write $P(m,p,x)$ for the probability of that state. The chemical master equation for the system is:

$$\begin{aligned}
\frac{\mathrm{d}P(m,p,x)}{\mathrm{d}t} = & -P(m,p,x)[r_{\mathrm{m}}p_{\mathrm{active}}(x) + \gamma_{\mathrm{m}}m + r_{\mathrm{p}}m + \gamma_{\mathrm{p}}p \\
& + k_{\mathrm{b,i}}\frac{c}{K_{\mathrm{b,i}}+c}\mathrm{p} + k_{\mathrm{b,e}}\frac{x}{K_{\mathrm{b,e}}+x}p + k_{\mathrm{a}}\frac{x}{K_{\mathrm{a}}+x}p \\
& + k_{\mathrm{nup}}c + k_{\mathrm{nup}}\xi x] \\
& + P(m+1,p,x)\gamma_{\mathrm{m}}(m+1) \\
& + P(m-1,p,x)r_{\mathrm{m}}p_{\mathrm{active}}(x) \\
& + P(m,p+1,x)\gamma_{\mathrm{p}}(p+1) \\
& + P(m,p-1,x)r_{\mathrm{p}}m \\
& + P(m,p,x+1)[k_{\mathrm{b,e}}\frac{x+1}{K_{\mathrm{b,e}}+x+1}p \\
& + k_{\mathrm{a}}\frac{x+1}{K_{\mathrm{a}}+x+1}p + k_{\mathrm{nup}}\xi(x+1)] \\
& + P(m,p,x-1)[k_{\mathrm{b,i}}\frac{c}{K_{\mathrm{b,i}}+c}p + k_{\mathrm{nup}}c]
\end{aligned} \tag{4}$$

Here, all the parameters of course have to be measured in counts, not in concentrations. As can be seen from the equation, none of the propensities depends explicitly on time and hence, we can use the hybrid algorithm between classical Gillespie and τ-leaping that we relied on.

Note that in the case with bursts that is discussed above, the transcription term $r_{\mathrm{m}}p_{\mathrm{active}}(x)P(m-1,p,x)$ is changed to $r_{\mathrm{m}}p_{\mathrm{active}}(x)\sum_{k=1}^{10}P(m-k,p,x)\frac{\theta\cdot(1-\theta)^{k-1}}{Z}$ with $Z=\sum_{k=1}^{10}k\cdot\theta\cdot(1-\theta)^{k-1}$ and $\theta=1/4$ in our example.

E Experimental materials and methods

Construction of $\Delta xapABR$ deletion strain. Cells containing plasmid pSIM6 were diluted 1:100 in 50 mL LB media and grown to an OD600 of ≈ 0.4 at 30°C. The culture was immediately placed in a water bath shaker at 43 °C for 15 minutes and then cooled in an ice bath for 10 minutes. Cells were then spun down for 10 minutes (4,000 g, 4 °C) and resuspended on ice in 50 mL of chilled water. This was repeated three times before resuspending in 200 µL of chilled water to generate competent cells. Homologous primer extension sequences were obtained from Baba *et al.* [18] and used to generate linear DNA containing a kanamycin resistance gene insert by PCR (amplified from plasmid pKD4), using primers that contained homology for the region on the chromosome to be deleted [19]. Electroporation was performed using 1 µL purified PCR product (about 100 ng DNA), mixed with 50 µL cells. Cells were resuspended in 750 µL SOC media and outgrowth was performed on a shaker at 30 °C for 90-120 minutes. Cells were then selected on an LB-agar plate containing kanamycin (30 µg/mL) and grown overnight at 30 °C. The deletions were confirmed by both colony PCR and sequencing. After confirmation, the deletion was transferred to a clean strain through P1 transduction and selection on kanamycin. Removal of kanamycin resistance was achieved by transforming cells with plasmid pCP20 (expressing FLP recombinase, carbenicillin resistance, on a temperature-sensitive replicon), with cells grown at 30 °C. Lastly, several colonies were then growth colony-purified once non-selectively at 43 °C and then tested for loss of antibiotic resistance for both kanamycin and carbenicillin.

Construction of $\Delta xapAB$ strain. Further experiments are underway in which precise control of the xapR copy number are needed and the preliminary data from these experiments are shown in the right panel of Fig. 2A of the main text. This strain was constructed by creating a XapR-mCherry fusion protein expressed from a tetR regulated promoter. This fusion construct was integrated into the ycbN locus of the $\Delta xapABR$ strain making an effective $\Delta xapAB$ strain. A pZS3-PN25-TetR expression plasmid was also inserted into this genetic background which permits titratable control over the xapR-mCherry copy number. For the data presented in this work, expression was maximized using 10 ng/mL of the TetR inducer anhydro-tetracycline. Further information of a similar construct (LacI-mCherry) can be found in [20] and [21].

Primer	Sequence	Comments
$xapABR <> P2$	actatttccggcaattcctgtctcctcac ctactgtgtcaatgcagccaaCATAT GAATATCCTCCTTAG	Forward primer for pKD4 with homology to *xapABR* locus
$xapABR <> P1$	agatacaacaggtatcgctaatatagagg aaagtgtcagagttaaaagatgtttt ttcatGTGTAGGCTGGAGCT-GCTTC	Reverse primer for pKD4 with homology to *xapABR* locus
xapABR-seq-fwd	cgggtcgttagctcagttggta	upstream of *xapR*, to check *xapABR* deletion
xapABR-seq-rev	ttcccagtcgaagaacccgtac	downstream of *xapA*, to check *xapABR* deletion

Table 2. Primers for deletion strains. Lower case sequences are homologous to a chromosomal locus. Upper case indicates sequences used for PCR amplification.

References

1. Phillips R, Belliveau NM, Chure G, Garcia HG, Razo-Mejia M, Scholes C. Figure 1 Theory Meets Figure 2 Experiments in the Study of Gene Expression. arXiv e-prints. 2018; p. arXiv:1812.11627.

2. Razo-Mejia M, Barnes SL, Belliveau NM, Chure G, Einav T, Lewis M, et al. Tuning Transcriptional Regulation through Signaling: A Predictive Theory of Allosteric Induction. Cell Systems. 2018;6(4):456 – 469.e10. doi:10.1016/j.cels.2018.02.004.

3. Norholm MH, Dandanell G. Specificity and topology of the *Escherichia coli* xanthosine permease, a representative of the NHS subfamily of the major facilitator superfamily. Journal of bacteriology. 2001;183(11466294):4900–4904.

4. Kaback HR. A chemiosmotic mechanism of symport. Proceedings of the National Academy of Sciences. 2015;112(5):1259–1264. doi:10.1073/pnas.1419325112.

5. Kaczorowski GJ, Robertson DE, Kaback HR. Mechanism of lactose translocation in membrane vesicles from *Escherichia coli*. 2. Effect of imposed $\Delta\Psi$, Δ pH, and $\Delta\mu_{\mathrm{H}^+}$. Biochemistry. 1979;18(17):3697–3704. doi:10.1021/bi00584a010.

6. Viitanen P, Garcia ML, Kaback HR. Purified reconstituted lac carrier protein from *Escherichia coli* is fully functional. Proceedings of the National Academy of Sciences of the United States of America. 1984;81(6324209):1629–1633.

7. Dandanell G, Szczepanowski RH, Kierdaszuk B, Shugar D, Bochtler M. *Escherichia coli* Purine Nucleoside Phosphorylase II, the Product of the xapA Gene. Journal of Molecular Biology. 2005;348(1):113–125.

8. Koszalka GW, Vanhooke J, Short SA, Hall WW. Purification and properties of inosine-guanosine phosphorylase from *Escherichia coli* K-12. Journal of bacteriology. 1988;170(3042752):3493–3498.

9. Li GW, Burkhardt D, Gross C, Weissman J. Quantifying Absolute Protein Synthesis Rates Reveals Principles Underlying Allocation of Cellular Resources. Cell. 2014;157(3):624 – 635. doi:https://doi.org/10.1016/j.cell.2014.02.033.

10. Milo R, Phillips R. In: Cell biology by the numbers. Garland Science; 2016. p. 217.

11. Razo-Mejia M, Phillips R. First-principles prediction of the information processing capacity of a simple genetic circuit. bioRxiv. 2019;doi:10.1101/594325.

12. Dornmair K, Overath P, Jähnig F. Fast measurement of galactoside transport by lactose permease. The Journal of biological chemistry. 1989;264:342–6.

13. Wright J K, Seckler R. The lactose/H+ carrier of *Escherichia coli*: lac YUN mutation decreases the rate of active transport and mimics an energy-uncoupled phenotype. The Biochemical journal. 1985;227:287–97. doi:10.1042/bj2270287.

14. Komatsu Y, Tanaka K. Deoxycytidine uptake by isolated membrain vesicles from *Escherichia coli* K 12. Biochimica et Biophysica Acta (BBA) - Biomembranes. 1973;311(4):496–506.

15. Munch-Petersen A, Mygind B, Nicolaisen AK, Pihl NJ. Nucleoside transport in cells and membrane vesicles from *Escherichia coli* K12. The Journal of biological chemistry. 1979;254 10:3730–7.

16. Wei C, Pohorille A. Permeation of Nucleosides through Lipid Bilayers. J Phys Chem B. 2011;115(13):3681–3688. doi:10.1021/jp112104r.

17. Golding I, Paulsson J, Zawilski SM, Cox EC. Real-Time Kinetics of Gene Activity in Individual Bacteria. Cell. 2005;123(6):1025–1036. doi:10.1016/j.cell.2005.09.031.

18. Baba T, Ara T, Hasegawa M, Takai Y, Okumura Y, Baba, M, et al. Construction of *Escherichia coli* K-12 in-frame, single-gene knockout mutants: the Keio collection. Molecular Systems Biology, 2006;2(1), 2460. doi:10.1038/msb4100050

19. Datsenko KA, Wanner BL. One-step inactivation of chromosomal genes in Escherichia coli K-12 using PCR products. PNAS, 2000;97(12), 6640–6645. doi:10.1073/pnas.120163297

20. Jones, DL, Brewster, RC, Phillips, R Promoter architecture dictates cell-to-cell variability in gene expression. Science, 2014;346(6216), 1533–1536. doi:10.1126/science.1255301

21. Chure G, Kaczmarek ZA, Phillips R. Physiological Adaptability and Parametric Versatility in a Simple Genetic Circuit. bioRxiv. 2019;doi:10.1101/2019.12.19.878462.
