# Supplementary material for: Theoretical investigation of a genetic switch for metabolic adaptation
Source: PLoS One. 2020 May 7;15(5):e0226453. doi: 10.1371/journal.pone.0226453 (PMC7205307; doi:10.1371/journal.pone.0226453)
Supplement: Attachment — Submitted filename: Reply to Reviewers.pdf [file pone.0226453.s002.pdf]

Response to Reviewer for PONE-D-19-32846 “Theoretical Investigation of a Genetic Switch for Metabolic Adaptation”

Kathrin S. Laxhuber[1], Muir J. Morrison[2], Griffin Chure[3], Nathan M. Belliveau[4], Charlotte Strandkvist[5], Kyle L. Naughton[6], Rob Phillips[2,3†]

[1] Department of Chemistry and Applied Biosciences, ETH Zurich, 8093 Zurich, Switzerland, [2] Department of Physics, California Institute of Technology, Pasadena, CA, USA, [3] Division of Biology and Biological Engineering, California Institute of Technology, Pasadena, CA, USA, [4] Howard Hughes Medical Institute, University of Washington, Seattle, WA, USA, [5] Department of Systems Biology, Harvard Medical School, Boston, MA, USA, [6] Department of Physics and Astronomy, University of Southern California, Los Angeles, CA, USA, [†] E-mail: phillips@pboc.caltech.edu

Author Response: Executive Summary

We thank the editor and reviewer for considering our manuscript and bringing some important points to our attention. The reviewer’s comments significantly improved our work and inspired us to make very interesting comparisons between the deterministic and stochastic picture of our system. Furthermore, as rightly suggested by the reviewer, we have now added experimental data to the manuscript and expanded the author list to reflect this. Below, we address the reviewer’s concerns one-by-one and indicate where changes were made either to the main text or the supporting information. Besides these changes, we made a few small improvements and corrections to the text that had slipped our attention before. We believe we have resolved the three issues brought up by the reviewer and hope our manuscript is now ready for publication in PLOS ONE.

Reviewer #1

Reviewer Comment

In their manuscript ”Theoretical investigation of a genetic switch for metabolic adaptation”, Laxhuber and colleagues present a very nice theoretical analysis of the behavior of the *E. coli* xapABR system, using both deterministic and stochastic simulations to demonstrate that the system can show meaningful and important bistability driven by the activity of the membrane transporter. In general the work is well done, and it is technically of high quality, but at present I have two substantial concerns that I think must be addressed in order for the paper to live up to its potential.

Author Response

We thank the reviewer for their careful reading of our work and are pleased that their impression of the manuscript is favorable overall.

Reviewer Comment

My first concern is that while the authors present their deterministic simulations first, and draw many of their conclusions primarily from those simulations, it seems to me that the use of such simulations is contra-indicated by the authors’ own findings. As both the number of XapR molecules and mRNA molecules are small integers (likely 10 or less), treatment of these species as continuous, deterministic quantities can never be more than a poor approximation. I find this troublesome for a couple of reasons. First, the authors still treat their continuous treatment as an apparently ’good’ way to model a genetic switch like that at work at the xapAB promoter; while I find their analysis insightful, in the end I cannot support such a recommendation given the stochasticity intrinsic in the low protein and mRNA copy numbers present here.

Author Response

We agree with the concern of the reviewer that in general, deterministic approximations of systems with such low copy numbers need to be verified. We do not intend to give the general recommendation that a continuous treatment alone is usually sufficient, and in order to avoid this, we added some words of warning in the beginning of our deterministic as well as our stochastic analysis.

Nevertheless, for our system we did successfully test the concordance of the stochastic simulations and the deterministic model. To make this more apparent, we included some additional comparisons of the two models. We address these in more detail in our responses below, but to summarize: we added a bifurcation diagram (Fig R1) which illustrates that the stochastic distributions track the deterministic fixed points very well, and we compared the stochastic timescales with the solution of the deterministic model (Fig R2) and found excellent agreement there too.

Even though stochastic fluctuations around the deterministic trajectories may be large, the stochastic trajectories do still flow to the fixed points as predicted by the deterministic model. Hence, the excellent agreement may be surprising or unintuitive at first, but the conclusions we draw from the deterministic phase diagrams turn out to be accurate.

Given this concordance, we prefer to start by showing the deterministic case and illustrating our conclusions there before moving on to the stochastic picture. That is because the deterministic phase diagrams have the advantage of providing the reader with a more intuitive understanding of the system than the plots of the results from the stochastic simulation.

Reviewer Comment

Second, while the authors do technically recapitulate the bistable behavior of the system in the stochastic version, the bistability seems based on their description to be quite fragile and not very meaningful, since it only occurs here for small distances between the lower and upper fixed points

Author Response

We thank the reviewer for bringing this point to our attention, which we believe to be a misunderstanding that resulted from a poor explanation in our original text. We believe we have clarified this in the manuscript, and allow us elaborate here too: Stochastic bimodality occurs only when two of the three fixed points are close together, or in other words, when the system is poised "sufficiently near" one of the bifurcation points. This may become clearer when looking at the bifurcation diagram that we added to our section about hysteresis at the end of the main text. For convenience, we insert this plot below in Fig R1 which addresses what we believe the reviewer is referring to: only when two of the three fixed points are close do the stochastic trajectories occupy both fixed points, resulting in bimodality.

Since this bimodality only occurs for a small region in parameter space, this actually indicates strong and not fragile bistability. In other words, the fact that the system does not stochastically "jump" to the other fixed point means that the fixed points are very stable to stochastic fluctuations. Thus, the switch-like feature is robust: there are two distinct states that the system can occupy, and only near the bifurcation points is it likely for stochastic trajectories to transition between them. Put differently, deterministic bistability does not need to result in stochastic bimodality, and in fact stochastic bimodality is contrary to robust bistability.

Reviewer Comment

(and seems to take an unreasonably long time to switch between states).

Author Response

To address the indeed valid point of unreasonable switching times, we have compared the timescales

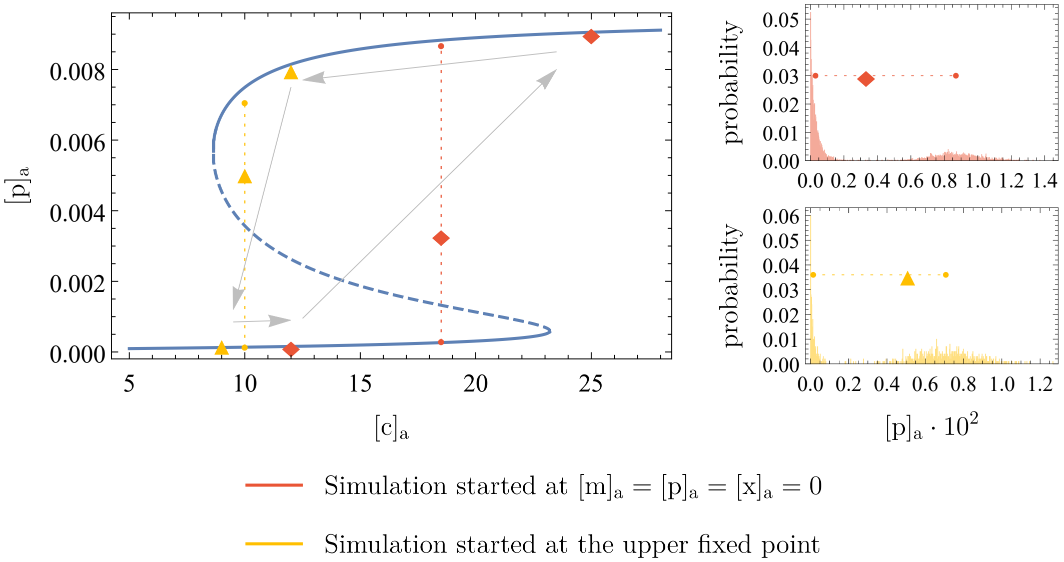

Figure R1. Bifurcation diagram showing the hysteresis loop in the stochastic system. The blue line shows the positions of the deterministic fixed points, while the dashed line indicates the instability of the middle one. The orange and yellow points show the mean of the stochastic simulations when started at zero and at the high fixed point, respectively. The positions of the two peaks in the bimodal distributions are indicated by smaller points, connected by dotted lines. To make this clearer, the bimodal distributions themselves are shown on the right. The arrows illustrate the hysteresis loop in the stochastic simulations.

in our stochastic simulations with those from the numerical solution of the deterministic model. We find excellent agreement, as shown in an updated figure in the main text and also below in Fig R2. So, again perhaps surprisingly, stochasticity does not materially alter the mean switching time as computed from the deterministic picture. Even adding additional stochasticity through transcriptional bursting (discussed more below) does not alter the mean switching time, though it does broaden the distribution of times. It is possible that this broadening resolves the apparent paradox. Even if the mean switching time is quite long, a non-negligible fraction of a population of cells could nevertheless switch on a shorter time-scale, especially with inclusion of extra stochasticity beyond what we have explicitly considered.

Reviewer Comment

I think the authors are likely correct in their assertion that they underestimate the amount of noise in the system due to not including transcriptional and translational bursting, and that the transition rates would likely be faster, but this gives no idea of how *much* faster, and thus it seems premature to simply declare victory and move on.

Author Response

This is a very reasonable concern which we have investigated more thoroughly now. We added rather strong transcriptional bursting to our simulation and do not find qualitative changes in the results. In particular, fluctuations around the mean become larger and, accordingly, distributions broader. Furthermore, bimodality begins to appear for lower values of the extracellular xanthosine concentration. Very interestingly, the change in the *mean* transition time between the stable fixed points is negligible. However, the *variance* in this time across many runs of the simulation becomes larger and the *peak* of the distribution shifts to shorter times. We have included two new figures in

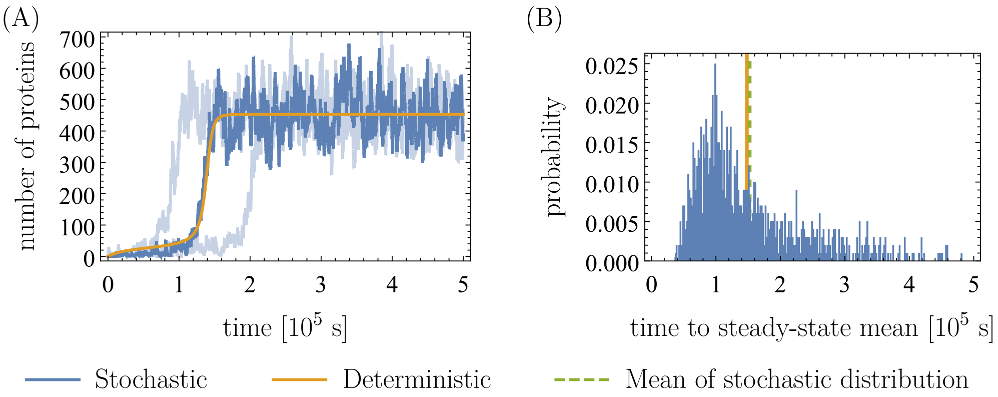

Figure R2. Stochastic and deterministic time evolution of protein (XapA/XapB) and adaptation time. (A) Blue shows the results from one typical run of the stochastic simulation, light blue shows two more extreme runs, and orange shows the trajectory obtained from solving the deterministic ODE's. The simulation was started at an mRNA, protein and intracellular xanthosine count of 0. The extracellular xanthosine concentration was chosen to be $[c]_a = 25$. (B) Blue shows the time until 90% of the protein concentration at the upper fixed point was reached in 1000 runs of the simulation (same conditions as (A)). The orange line shows the corresponding deterministic time. The green dashed line right next to it indicated the mean of the blue distribution.

the SI discussing these results (Figs. 6 and 7), along with discussion there and in the main text.

Reviewer Comment

My recommendation would be that the authors put in some more cautionary words about the applicability of the continuous model, and ideally spend a little more time characterizing the discrete model. in terms of switching times, parameters that give rise to bimodal behavior, effects of incorporating reasonable amounts of transcriptional bursting, etc. Some more direct comparisons of the behavior of the two models in similar ranges of parameters space would also likely be enlightening.

Author Response

We hope that we have addressed all these points in our responses above and shortly summarize here: We put in some cautionary words about the general usage of continuous models (beginning of sections on deterministic as well as stochastic results) and demonstrated that for our system and the conclusions we draw, the continuous model is surprisingly appropriate (bifurcation diagram and time evolution, end of results section). Furthermore, we extended our characterization of the stochastic model as suggested by investigating the distribution of adaptation/switching times (end of results section) as well as by incorporating transcriptional bursting in our simulation (end of section C in S1 Text). The deterministic and stochastic model are now directly compared (for the same set of parameters) in the bifurcation diagram as well as in terms of their adaptation/switching times.

Reviewer Comment

My other major difficulty here is that essentially all of the work is premised on reference 22, which is 'unpublished data.' This is the only experimental evidence given for the bistability of xapAB expression, justification of many of the model parameters, experimentally expected switching timescales, etc. It is impossible to properly interpret the present work, either for the purposes of review or from the viewpoint of an interested reader, without availability of those data, which either ought to be

their own independent publication or ought to be included with this one.

Author Response

With further consideration, we agree with the reviewer that we relied rather heavily on this unpublished data and that it is therefore essential to make the experimental data available. We have opted to include the data with this publication rather than separately. For this reason, we have added Griffin Chure, Nathan Belliveau, Charlotte Strandkvist, and Kyle Naughton to the author list, who collected or were involved in collecting the data. We have added a new section, "experimental motivation," in the beginning of the main text which discusses the main features of the data that are relevant for our model. Furthermore, we added a section on the materials and experimental methods at the end of S1 Text (section E). The raw data is publicly available at DOI: 10.5281/zenodo.3695049.
