# Supplementary material for: Theoretical investigation of a genetic switch for metabolic adaptation
Source: PLoS One. 2020 May 7;15(5):e0226453. doi: 10.1371/journal.pone.0226453 (PMC7205307; doi:10.1371/journal.pone.0226453)
Supplement: Attachment — Submitted filename: Response to Reviewers.pdf [file pone.0226453.s003.pdf]

Second Response to Reviewer for PONE-D-19-32846 "Theoretical Investigation of a Genetic Switch for Metabolic Adaptation"

Kathrin S. Laxhuber[1], Muir J. Morrison[2], Griffin Chure[3], Nathan M. Belliveau[4], Charlotte Strandkvist[5], Kyle L. Naughton[6], Rob Phillips[2,3†]

[1] Department of Chemistry and Applied Biosciences, ETH Zurich, 8093 Zurich, Switzerland, [2] Department of Physics, California Institute of Technology, Pasadena, CA, USA, [3] Division of Biology and Biological Engineering, California Institute of Technology, Pasadena, CA, USA, [4] Howard Hughes Medical Institute, University of Washington, Seattle, WA, USA, [5] Department of Systems Biology, Harvard Medical School, Boston, MA, USA, [6] Department of Physics and Astronomy, University of Southern California, Los Angeles, CA, USA, [†] E-mail: phillips@pboc.caltech.edu

Author Response: Executive Summary

We would like to thank the editor and reviewer for the thorough and very constructive review process. We are happy about the positive overall feedback and hope that we have now resolved any remaining ambiguities.

Reviewer #1

Reviewer Comment

In general the authors have done an admirable job addressing my comments.

Author Response

We thank the reviewer and are glad that we could clarify all previous issues.

Reviewer Comment

At this point, I find myself confused on one issue, which I hope the authors will address (unless I'm just being truly dense). In the right side of Figure 9, a series of phase portraits are displayed. I assume that these must have been from the deterministic system, since the authors make no reference to performing a suitable scan of conditions to obtain such diagrams for the stochastic system; I assume that these in fact arise from the deterministic system. However, the figure legend does not address this point at all, and the reader is left with the impression that those phase portraits arise from the stochastic system. The issue is not helped by the fact that the coloring and axis scaling for the right side of Figure 9 does not follow the same conventions used in earlier diagrams; for example, the right panel of Fig 9A looks a lot like Fig. 5B, but the colors and axis scales are different.

Author Response

This is a very good point that we had missed, and we thank the reviewer for reading our manuscript so carefully. The phase portraits are indeed from the deterministic system. We now addressed this in the figure legends and have changed the coloring in Fig 9.

Reviewer Comment

I would also like to caution the authors (and editorial staff) that while the "Revised Manuscript with Track Changes" document was fully readable, the figure numbering of the main text figures seemed to be off in the submission system.

Author Response

The reason behind this apparent problem is that in the PDF, the figure numbering starts at one, while it starts at zero in the figure filenames. Thus, there is a shift of one between the figure filenames and the numbering in the PDF. However, the names and numbering in the original tex file code correspond to the figure filenames, such that we believe it should be clear to the editorial staff whenever they include the figures in the tex file.

Reviewer Comment

As a final note, I would like to urge the authors to make the source code for their simulations publicly accessible, which would greatly help the community in adapting similar models for other systems.

Author Response

We fully agree with the reviewer and therefore have made all source code publicly available in the same repository as the raw data: `https://doi.org/10.5281/zenodo.3733595`.
